# Spectral Heterogeneity of Thioflavin T Binding to Aβ42:Aβ40 Mixed Fibrils: Implications for Alzheimer’s Disease Screening

**DOI:** 10.1021/acsomega.5c02756

**Published:** 2025-04-21

**Authors:** Kiyo Fukase, Akane Iida-Adachi, Hideki Nabika

**Affiliations:** † Graduate School of Science and Engineering, Yamagata University, 1-4-12, Kojirakawa, Yamagata 990-8560, Japan; ‡ Faculty of Science, Yamagata University, 1-4-12 Kojirakawa, Yamagata 990-8560, Japan

## Abstract

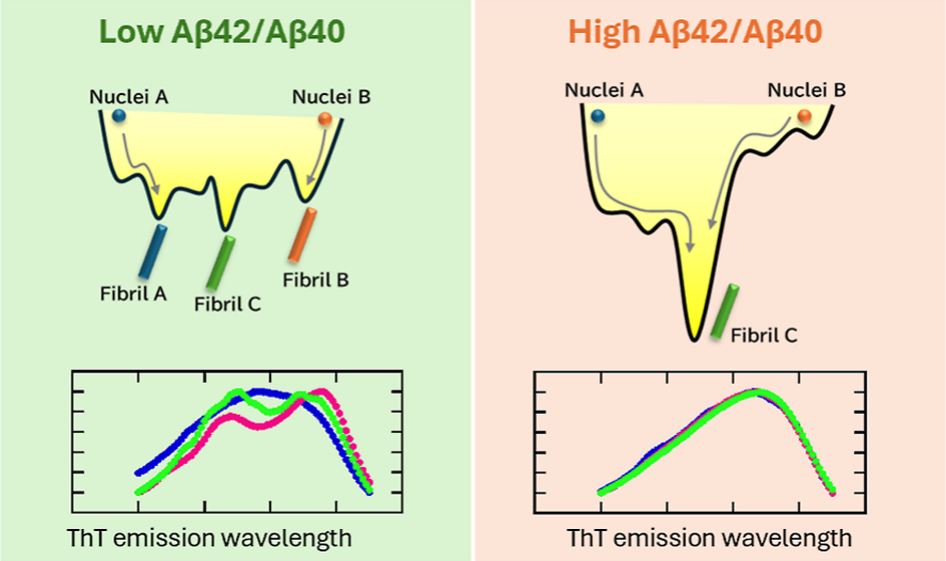

In Alzheimer’s disease (AD), the amyloid β (Aβ) protein self-assembles, whereby Aβ40 and Aβ42 peptides interact, forming a mixed fibrillar assembly. Evaluating local Aβ40:Aβ42 mixed fibril conformations remains challenging, requiring a simple method to compare microscopic (molecular-scale) and macroscopic (plaque-scale) findings. The aim of the current study was to design a method to analyze Aβ fibril formation in a single sample without drying via fluorescent thioflavin T (ThT) labeling. The analysis revealed spectral heterogeneity associated with the ThT-binding mixed fibrils. Although the fluorescence wavelength associated with higher Aβ42:Aβ40 fibril ratios remained relatively unchanged, those associated with lower Aβ42:Aβ40 fibril ratios exhibited significant heterogeneity. This suggests that the local β-sheet structure exhibits significant variability at lower Aβ42:Aβ40 ratios. This specific feature can be attributed to differences in the shape of the “funnel” in the energy landscape during Aβ assembly. Thus, our protocol facilitates rapid and efficient screening of fibril conformational alterations compared to conventional techniques. Cumulatively, our results demonstrate that comparing the spectral features of ThT with the kinetic and morphological characteristics of a single sample provides specific molecular insights related to the origin of Aβ42:Aβ40 ratio-dependent molecular mechanism—insights that cannot be detected through conventional kinetic and morphological analyses alone.

## Introduction

Molecular self-assembly of amyloid β (Aβ) proteins, derived from amyloid precursor protein (APP), into one-dimensional fibrils is a pathological hallmark of Alzheimer’s disease (AD). Monomeric Aβ proteins assemble into dimers, oligomers, protofibrils, fibrils, and amyloid plaques,^1,2^ according to the thermodynamic energy landscape.^3,4^ Given that the thermodynamic stability of the secondary, tertiary, and quaternary states depends on the primary structure of the protein, the assembly kinetics strongly depend on the primary monomeric Aβ protein structure cleaved from APP. There are two dominant monomeric Aβ isoforms, 40-residue Aβ40 and 42-residue Aβ42. While Aβ40 is more abundant, Aβ42 is more toxic, with higher hydrophobicity and fibrillogenicity.^5^ The normal physiological Aβ42:Aβ40 ratio in the brain is ∼1:9; however, in the brains of patients with familial AD, Aβ42 increases, leading to an Aβ42:Aβ40 ratio of 3:7,^6−8^ which is associated with increased synaptotoxicity and amyloid plaque formation. This change in the Aβ42:Aβ40 ratio from 1:9 to 3:7 affects aggregation kinetics, amyloid fibril morphology, and synaptic function, leading to increased neurotoxicity.^8^

The molecular role of Aβ42 in rapid fibril formation and aggregation has been evaluated using the thioflavin T (ThT) binding assay,^9−16^ transmission electron microscopy (TEM),^12−16^ atomic force microscopy (AFM),^17,18^ nuclear magnetic resonance (NMR),^10,12,15,16,19^ mass spectrometry,^11,12,20^ circular dichroism,^12,21^ fluorescence microscopy,^22^ IR spectrometry,^23^ and numerical simulations.^11,24,25^ Associated analyses have revealed that Aβ40 and Aβ42 self-assemble into a mixed oligomer with random distribution^23,26^ and an intermediate structure of pure oligomers.^23^ Because the primary structures of Aβ40 and Aβ42 differ, the kinetics of the assembly process may be strongly influenced by the incorporation of other Aβ proteins to form mixed oligomers. Furthermore, addition of Aβ monomers with residues that differ from those of Aβ42 or Aβ40 inhibit and deaccelerate fibril formation. This suggests that the structural mismatch between Aβ42 and Aβ40 is thermodynamically unfavorable for assembly. However, contradictory results have suggested that adding Aβ seeds with residues different from Aβ42 or Aβ40 monomers promotes and accelerates fibril formation in a concentration-dependent manner.^9,14^ This is because the nucleation step that should overwhelm the nucleation energy is eliminated by the presence of preformed fibrils.

To evaluate fibril formation kinetics, ThT is commonly employed as a probe for fluorescent evaluation of β-sheets.^27−32^ ThT is a molecular rotor that rotates around a single C–C bond between the benzothiazole moiety and dimethylaniline ring. In low-viscosity solvents such as water, a rapid shift occurs from the photoexcited state of ThT into the transient nonfluorescent relaxed ground state (i.e., twisted internal charge transfer (TICT) state) via rotation at the C–C bond.^33,34^ Hence, the increase in local viscosity or the insertion of ThT into confined spaces suppresses its rotational motion, reducing relaxation into the TICT state and restoring the emissive nature of ThT. Consequently, it has been reported that nonradiative relaxation pathways are minimized, leading to an increased quantum yield of ThT when intercalated between β-sheets that are orthogonal to the β-strands.^35^ Accordingly, because rotation is inhibited by Aβ fibril adsorption at the β-sheet site, it can be used as a fluorescent sensor to detect Aβ fibrils. Moreover, the transition energy, i.e., the excitation and emission wavelengths, is strongly dependent on the angle between benzthiazole and benzene rings,^28^ which highlights that the degree of confinement and local structure between β-sheets alters the excitation and emission wavelength of ThT through changes in rotational restriction. Thus, ThT is a valuable tool for evaluating Aβ monomer assembly into fibril form, revealing that Aβ42 fibrils form faster than Aβ40 fibrils.^14^

While the ThT assay provides quantitative information on the kinetics and quantity of Aβ fibrils formed from Aβ monomers, qualitative information is acquired through other methods such as TEM, AFM, and NMR.^10−16,36^ Although these methods help determine the residue-based influence of different Aβ proteins, sample preparation such as drying is required, which may alter Aβ fibril integrity. This can be mitigated by careful handling to retain the structural properties of Aβ fibrils dispersed in the solution phase. An ideal method would allow for quantitative and qualitative evaluation of the same sample without drying. Nevertheless, ThT holds promise as a sensor of Aβ fibril dynamics in terms of quantitative and qualitative aspects such as β-sheet stacking (i.e., TICT is sensitive to the local morphological nature of Aβ fibril β-sheets^37^). Indeed, ThT has been successfully employed to detect differences in local insulin fibril conformation, reinforcing its potential application for detecting other proteins, including Aβ, prions, and α-synuclein.^38^

In the current study, we sought to characterize Aβ fibril formation without drying by fluorescently labeling ThT and via kinetic and macroscopic analyses. Collectively, our results show that comparing the spectral properties of ThT with the kinetic and morphological aspects of the same solution sample yields information on the Aβ42:Aβ40 ratio-dependent structural difference, which cannot be detected solely with conventional kinetic and morphological techniques.

## Results and Discussion

**Figure 1:**
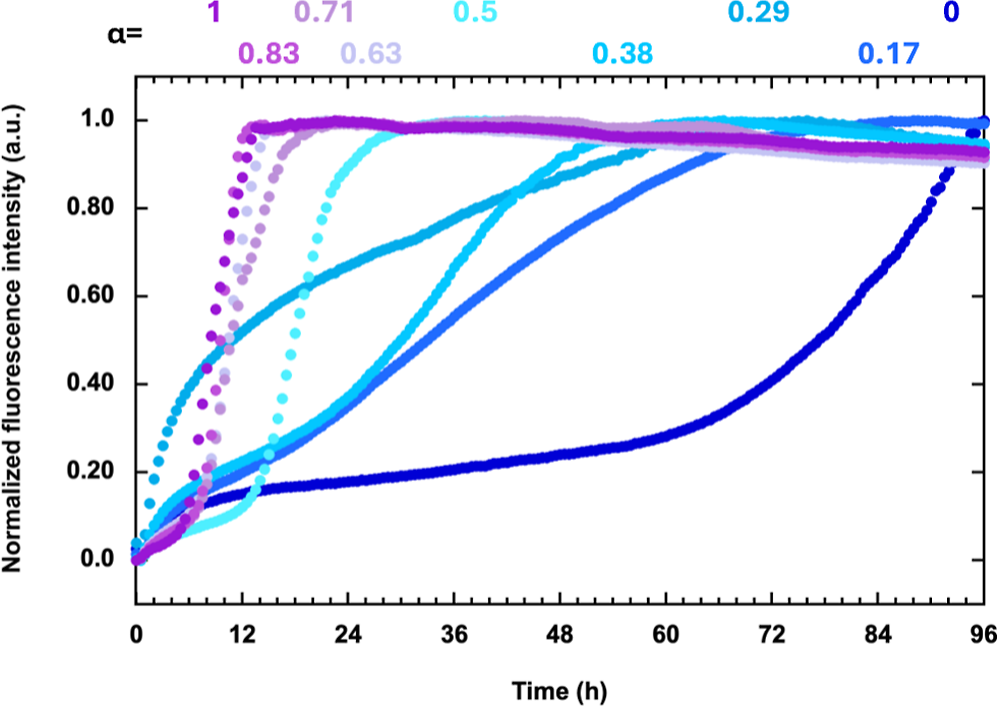
Kinetics of fibril formation at various α values.

Fibril formation kinetics were assessed using the ThT assay (Figure 1) at various α composition ratios. At α = 0 (the solution contained only Aβ40), the emission intensity of ThT gradually increased over 60 h, followed by rapid growth for 30 h. This indicates that nucleation occurred slowly during the 60 h induction period, after which fibril growth was triggered by the formation of nuclei. At α = 1 (the solution contained only Aβ42), fibrils formed rapidly within 12 h, without extended induction. The absence of the induction period indicates that Aβ42 nucleation occurred markedly faster than that of Aβ40, consistent with previous results.^11,14,36^ Under coexistence conditions (0 < α < 1), the fibril growth rate occurred in an α-dependent manner; fibril growth increased with increasing α. This suggests that Aβ42 accelerates Aβ40 fibril formation, whereas Aβ40 decelerates Aβ42 fibril formation. Most curves were comparable (normalized fluorescence intensity increased to ∼0.1–0.2) during the first 6 h; however, for the α = 0.29 sample, the fluorescence intensity increased rapidly to 0.4. Since the factors driving the deviations in the α = 0.29 sample were unclear, this sample was excluded from subsequent analyses.

**Figure 2:**
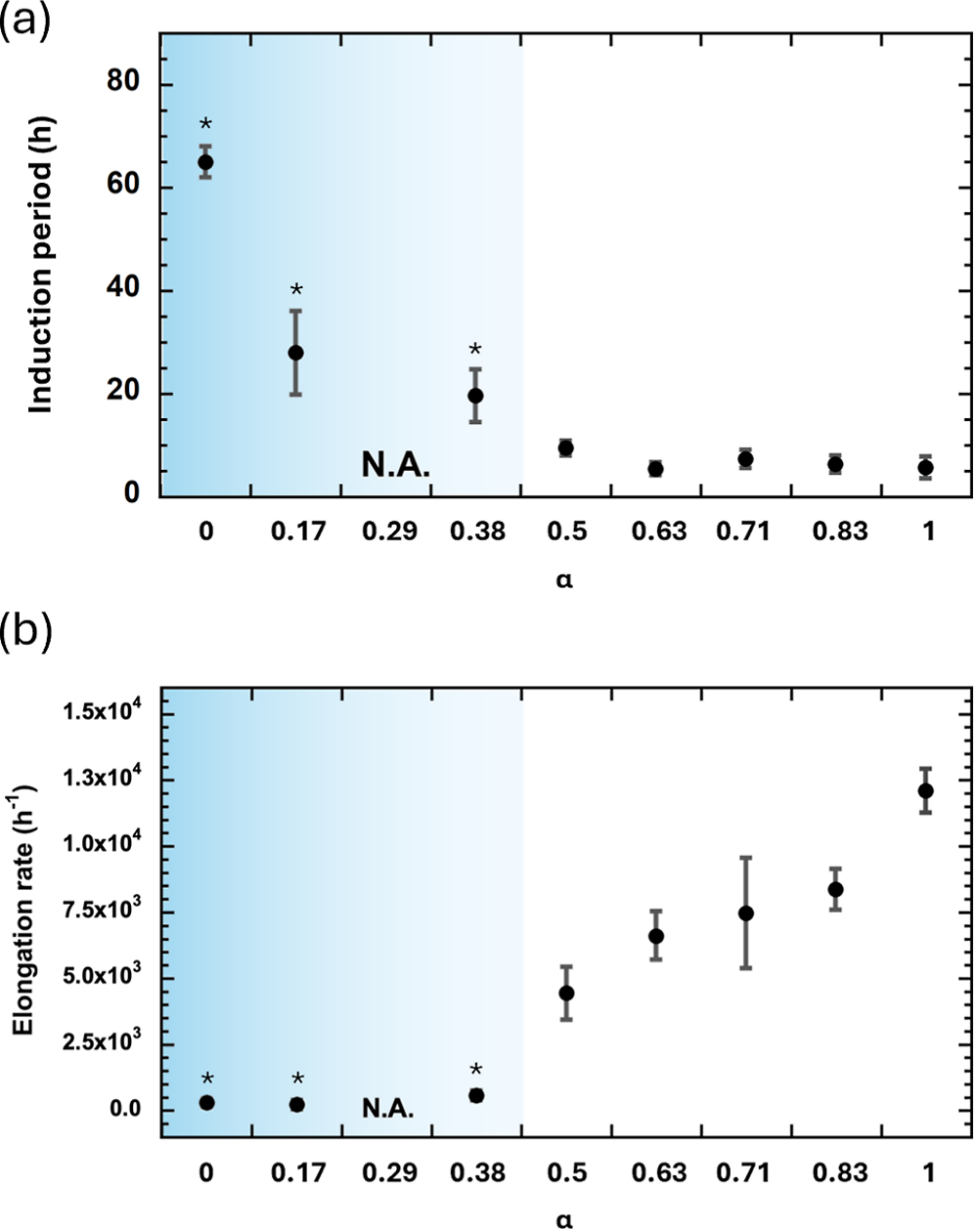
(a) Induction period and (b) elongation rate as a function of α, evaluated from the fibril growth curves. Data is represented as mean ± SD where *n* = 6. Data was analyzed via Mann–Whitney U test compared to data for α = 0.5. *p < 0.01.

To further quantitatively evaluate the fibril growth process, we estimated the induction period (Figure 2a) and elongation rate (Figure 2b) for each sample, excluding the α = 0.29 sample (Figure 1). The induction period decreased from 65 h for α = 0 (Aβ40 only) to 5 h for α = 1 (Aβ42). The α-dependent change was more evident for the lower α region; the induction was comparable for α > 0.5 and approximately equal to that of α = 1. This indicated that adding the Aβ40 monomer to the Aβ42 monomer did not significantly affect Aβ42 nucleation, whereas the addition of Aβ42 to Aβ40 accelerated the nucleation of homo- (Aβ40–Aβ40) and/or hetero- (Aβ40–Aβ42) nuclei. Additionally, the addition of the Aβ42 monomer formed Aβ42 homo- (Aβ42–Aβ42) nuclei that promoted the growth of oligomers and fibrils while consuming Aβ40 monomers. This was responsible for the observed reduction in the induction period.

A similar trend was observed for the elongation rate, where the α-dependent change differed between lower and higher α regions. The elongation rate was low and comparable for α < 0.38, indicating that the addition of Aβ42 to Aβ40 did not contribute to fibril growth. In contrast, addition of Aβ40 to Aβ42 (α > 0.5) effectively deaccelerated fibril growth in an α-dependent manner. Because the samples with α > 0.5 contain the same amount of Aβ42 (5 μM, see Table 1 in the experimental section), fibril growth would occur at the α = 1 rate. However, we have observed that with the addition of Aβ40, fibril growth decreased even when the concentration of Aβ42 was kept constant. This suggests that Aβ40 may be incorporated into Aβ42 fibrils; however, owing to differences in their primary structures, the formation of mixed fibrils creates an energy barrier that hinders the assembly of additional monomers, ultimately leading to a decrease in fibril growth.

**Table 1:** Final Concentrations of Each Component and Corresponding α-values for Nine Samples Measured in This Study

sample no	Aβ40 (μM)	Aβ42 (μM)	ThT (μM)	α
1	5	0	10	0
2	5	1	10	0.17
3	5	2	10	0.29
4	5	3	10	0.38
5	5	5	10	0.5
6	3	5	10	0.63
7	2	5	10	0.71
8	1	5	10	0.83
9	0	5	10	1

Based on the induction period and elongation rate data, the addition of Aβ monomers with different residues had the following effects: (1) under Aβ40-dominant conditions, nucleation was accelerated while fibril growth was not affected; (2) under Aβ42-dominant conditions, nucleation was not affected, but fibril growth decelerated. These differences between Aβ40-dominant and Aβ42-dominant conditions are likely due to differences in the native characteristics of Aβ40 and Aβ42. For instance, Aβ40 and Aβ42 have different energy landscapes and exhibit structural diversity at the nucleation step.^39^ Because the energetic and structural properties of the main component nuclei differ, it is reasonable that susceptibility to the second component also differs.

**Figure 3:**
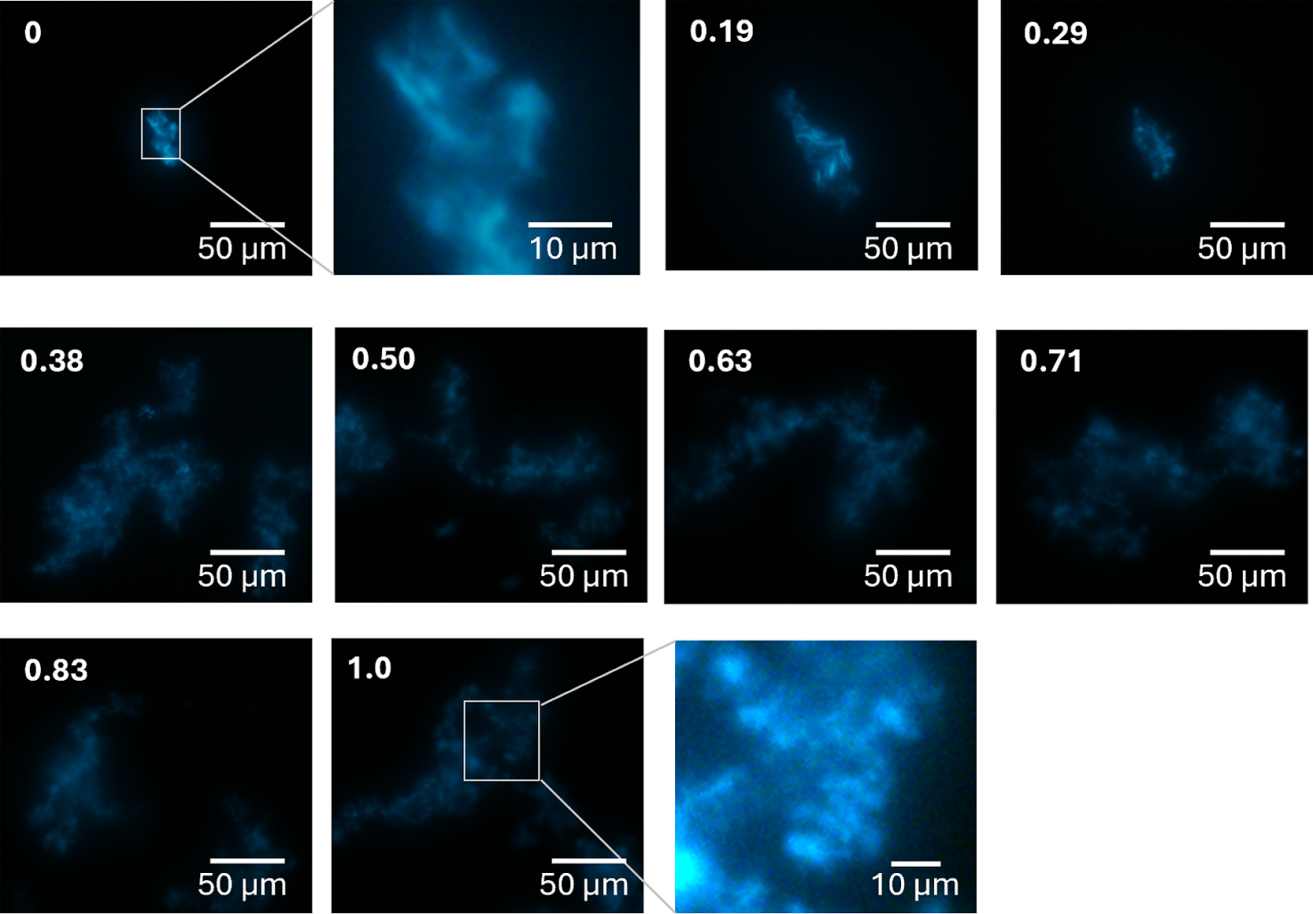
Fluorescence microscopy images of fibrils formed under each α-condition. Enlarged images are shown for α = 0 and α = 1. The brightness of the enlarged image for α = 1 was increased for improved clarity.

The macroscopic dependence of the samples was characterized using fluorescence microscopy. Under all conditions, bright blue aggregates emitted from fluorescent ThT were observed (Figure 3). However, aggregate brightness and size changed based on α: for α = 0, 0.17, and 0.29, the aggregate was compact and bright; at α = 0.38, the aggregate was larger with lower brightness. Further increases in α resulted in larger, duller aggregates; as α increased, the aggregates became dark and sparse. Although it is unclear whether there is any α-dependency, we observed rod-like aggregates (shown in the enlarged image for α = 0) and dot-like aggregates (shown in the enlarged image for α = 1.0). Depending on the assembly conditions, amyloid proteins can form crystal-like amyloid fibrils and glass-like aggregates.^40^ However, because both aggregate types were observed under fluorescence microscopy with ThT, a selective marker for β-sheet formation, both comprised crystal-like amyloid fibrils. Therefore, the difference between these aggregate types is due to the elongation differences of each fibril. That is, long fibers are likely to form long fibril aggregates, whereas short fibers are likely to form relatively structureless aggregates with small dot-like structures.

**Figure 4:**
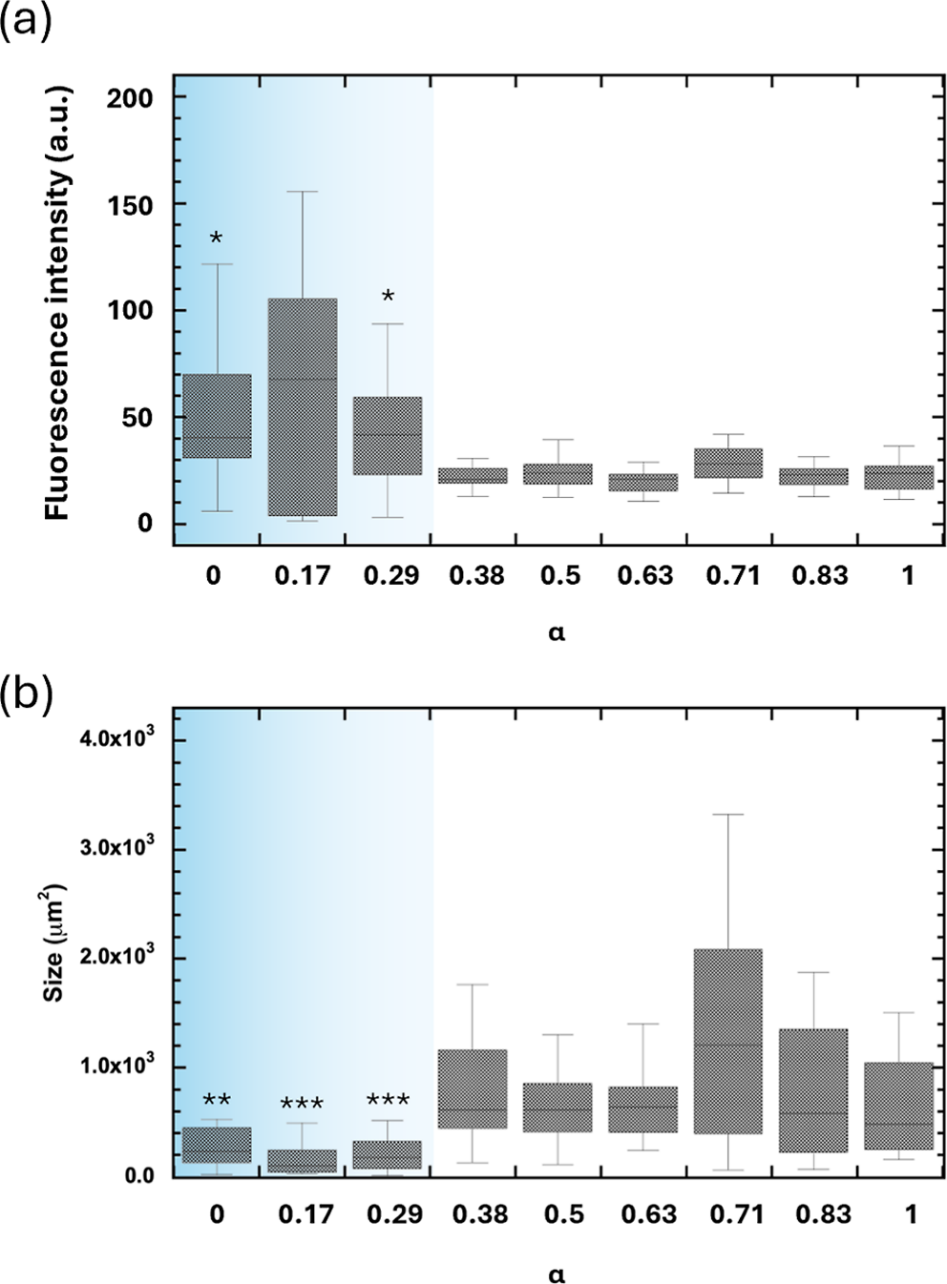
Variation in the (a) fluorescence intensity and (b) size of fibrils as a function of α. Data is represented as mean ± SD where *n* = 12–31 aggregates per group. Data was analyzed using the Mann–Whitney U test, with comparisons between data for α = 0.38. *p < 0.05, **p < 0.001, ***p < 0.0001.

Quantitative analysis of these α-dependent macroscopic characteristics was performed using imaging software (Figure 4). For the lower α condition, the fluorescence intensity was approximately 50, and the size was <500 μm^2^; however, some aggregates exhibited a fluorescence intensity >100. In contrast, for the higher-α conditions, fluorescence intensity decreased by approximately 50%, while the size more than doubled. As observed in the microscopy images, these numerical data confirmed that the aggregates were bright and compact at lower α conditions, and dark and larger at higher α conditions. The fluorescence intensity is dependent on the (i) density of ThT adsorbed on fibrils, (ii) fluorescence quantum yield of ThT adsorbed on fibrils, and (iii) density of fibrils in each aggregate. Considering that molecular-scopic^25,41,42^ and microscopic^18,36^ features have been reported to differ between Aβ42 and Aβ40, it is plausible that the local structure of β-sheet stacking differs, making possibilities (i) and (ii) probable if the β-sheet stacking structure, which serves as the host site for ThT, changes in an α-dependent manner. Specifically, in possibility (ii), it has been reported that the fluorescence intensity of ThT adsorbed on Aβ40 fibrils is 1.7 times higher than that of ThT adsorbed on Aβ42 fibrils.^43^ Our results, which showed a 2-fold higher fluorescence intensity under Aβ40-rich conditions (α < 0.29), support that the fibrils are likely of Aβ40 origin, whereas those at α > 0.38 are likely of Aβ42 origin.

**Figure 5:**
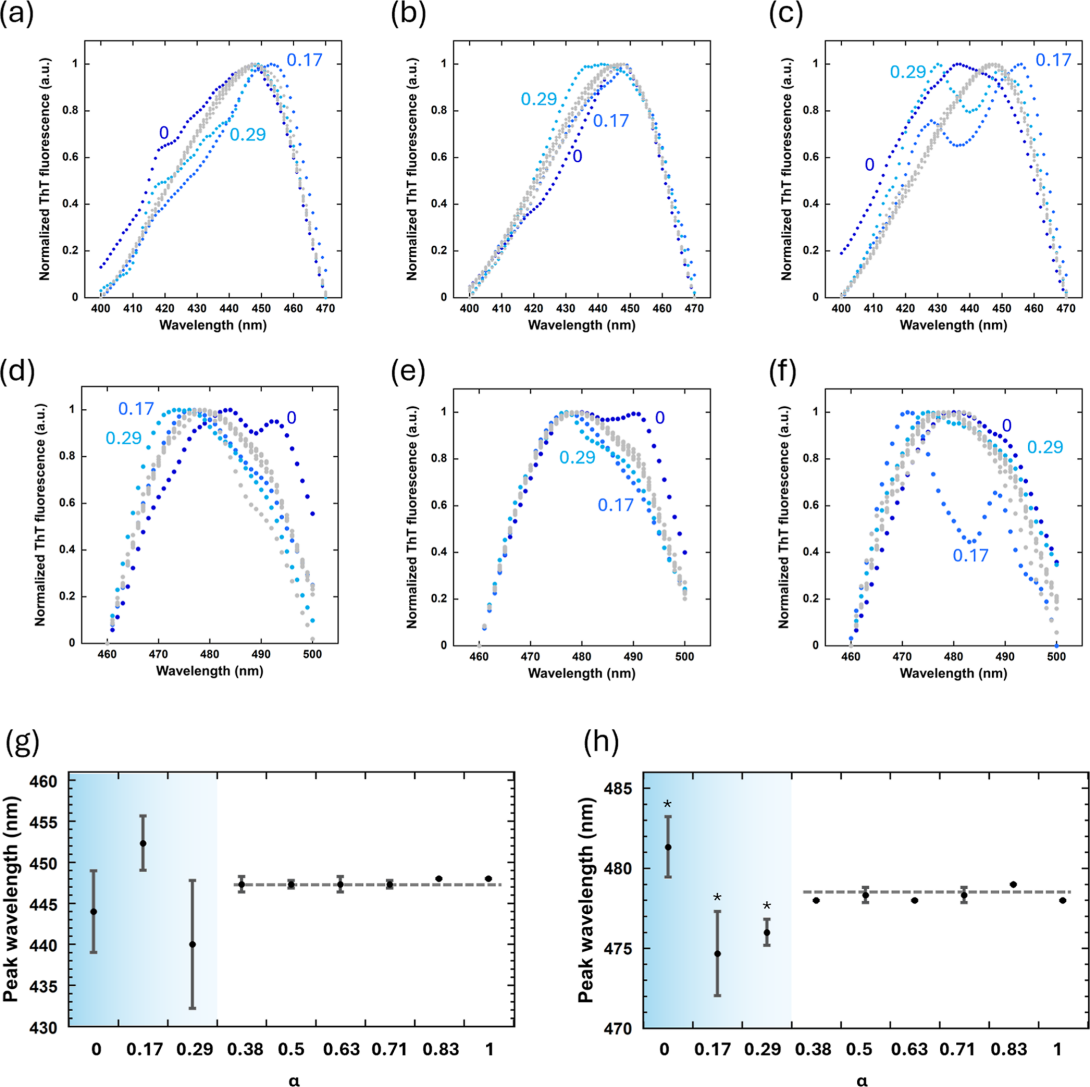
Excitation (a–c) and emission (d–f) spectra for three measurement sets. Excitation spectra in each figure represent one of the triplicate experiments. Spectra for samples with α > 0.38 are presented in gray. Peak wavelengths of excitation (g) and emission (h) spectra as a function of α. Data is represented as mean ± SD where *n* = 3. Data was analyzed using Mann–Whitney U test, with comparisons between data for α = 0.38. *p < 0.05.

To gain further insight into the local structure of β-sheets without a drying process, the excitation and emission spectra of ThT were acquired (Figure 5). It should be noted that the emission spectra primarily reflect ThT molecules interacting with fibrils, as unbound ThT is nonemissive, which is confirmed by the negligible fluorescence at time = 0 in the ThT assay curves (Figure 1). Thus, far, we observed that both the kinetic (Figure 2) and macroscopic (Figure 4) data exhibited similar trends in lower and higher α regions. The induction period demonstrated a monotonous decrease followed by a constant phase (Figure 2a), while the elongation rate remained relatively constant, followed by a monotonous increase (Figure 2b). Furthermore, the fluorescence intensity was high and low in lower- and higher-α regions, respectively (Figure 4). Similar to these results, the emission properties of ThT differed between high and low α conditions. Most spectra for samples with α > 0.38 overlapped at an excitation maximum of 450 nm (Figure 5a), indicating that the excitation spectra for these samples were relatively similar. In contrast, the excitation spectra of the samples with α < 0.29 deviated from those with α > 0.38. Relative intensities at shorter wavelengths were lower for α = 0.29 and 0.17 and higher for α = 0. Furthermore, the peak wavelength exhibited a blueshift for α = 0.17. The tendency for the spectra to overlap for α > 0.38 and deviate for α < 0.29 was confirmed in the remaining two measurements (Figure 5b,c). These results indicate that the emission properties of fibril-bound ThT are almost identical under high α conditions, whereas under low α conditions, a variety of ThT molecules with different emission properties are likely present.

Similar trends were observed for the emission spectra (Figure 5d–f); the spectra were relatively identical, with a single peak at 480 nm for α > 0.38. In contrast, the spectra for the samples with α < 0.29 deviated and occasionally showed spectra with shifted double or shoulder peaks. Similar to the excitation spectra, various emission properties of ThT were observed, with unique shapes for different samples. These characteristic features for the higher- and lower-α regions become more evident by comparing the dependence of the peak wavelengths on α. For excitation (Figure 5g) and emission (Figure 5h), the peak positions were comparable for α > 0.38, with small differences between the experiments. In contrast, the peak excitation and emission wavelengths exhibited large variations, with no monotonous tendency, for α < 0.29.

**Figure 6:**
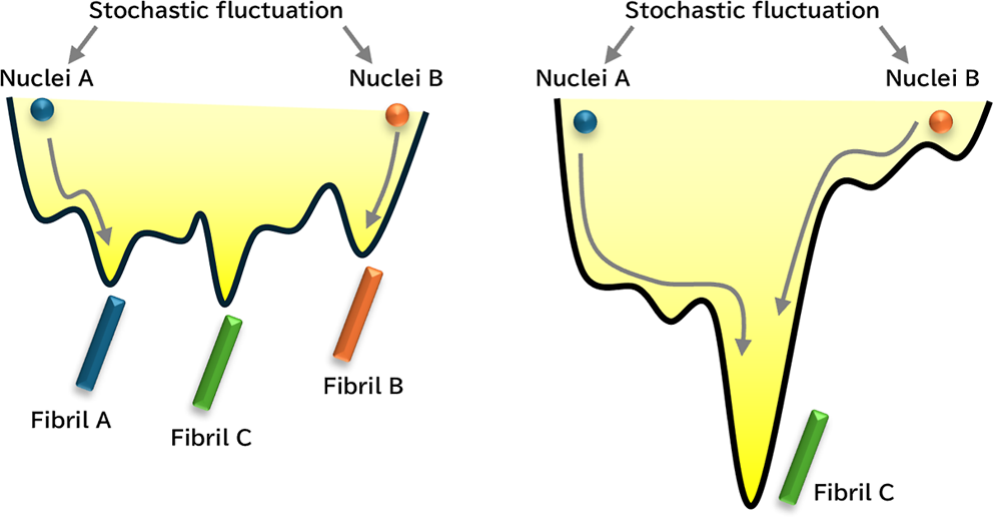
Schematic illustrations of (a) multiple shallow funnel and (b) a single deep funnel models.

The emission properties of ThT depend on the local environment of β-sheets.^37^ Due to its high molecular weight and its flexibility, Aβ monomers assemble into a variety of polymorphic nuclei, oligomers, protofibrils, and fibrils.^44−46^ The initial step in these polymorphisms is the nucleation, where Aβ monomers form various polymorphic nuclei in a stochastic process (shown as Nuclei A and Nuclei B in Figure 6). When a deep local minimum exists, both nuclei settle into this deep minimum regardless of stochastic fluctuations in the initial nucleation step (right panel in Figure 6). However, if there are multiple energy minima of similar depth within the same vicinity, stochastic fluctuations during nucleation can lead to the formation of different fibrils (left panel in Figure 6). As mentioned earlier, the fluorescence wavelength of ThT varies depending on the state of β-sheets; therefore, fibrils in different energy minima exhibit distinct ThT fluorescence properties. The fact that only a single fluorescence property was observed under higher α-conditions, while multiple fluorescence properties were found under lower α-conditions, suggests that fibril growth follow a single deep funnel model (right panel) under higher α-conditions, and a multiple equivalent funnel model (left panel) under lower α-conditions. This explains the α-dependent fluorescence properties observed in our system. Notably, structural peculiarities under lower α conditions have been previously reported through TEM observations, where fibrils aligned with those under higher α conditions; however, with observed twisting.^8^

Since small differences in local steric structure can culminate in differences in macroscopic fiber structure, the peculiar fluorescence outcomes of ThT may indicate the structural peculiarities specific to low α conditions. As this composition region mimics those in patients with familial AD, our results showing diversity in β-sheet structure could be associated with the specific molecular mechanism in the brain. However, such differences in microscopic structure cannot be detected using conventional techniques such as TEM, but rather require fluorescence spectrometry. This highlights the potential of the described method for the rapid and efficient screening of the microscopic structure of fibers across various contexts, including clinical settings.

All the experiments were conducted with varied total Aβ concentration, as shown in Table 1 in the experimental section. However, it is well established that the reaction kinetics of each elementary step such as nucleation and fibril elongation are dependent on Aβ concentration. Therefore, it would be valuable to conduct additional experiments with varying α and fixed Aβ concentrations, and comparing the results with our current findings. To further this aim, we conducted an additional experiment with the total protein concentration fixed at 5 μM, while all other experimental conditions and procedures remained unchanged. Overall, the data (kinetics, morphology, and ThT emission) exhibited similar trends to the original findings (see the Supporting Information), with the kinetics, morphology, and ThT emission properties differing between low and high α conditions, regardless of whether the total concentration was fixed.

## Conclusion

The kinetics of fibril formation, macroscopic characteristics of fibrils, and fluorescent nature of ThT bound to fibrils were evaluated for the same samples in an aqueous phase using ThT as the β-sheet marker. The obtained data were categorized into two regions, with an α boundary of ∼ 0.3–0.4. Compared to single-component systems (α = 0 and 1), under Aβ40-dominant conditions, nucleation accelerated, while fibril growth remained unaffected. In contrast, under Aβ42-dominant conditions, nucleation was not affected, whereas fibril growth decelerated. Fluorescence microscopy observations revealed that under the lower α condition, bright and compact aggregates formed, while under the higher α condition, dark and larger aggregates were observed. The fluorescence spectrum for the lower α region exhibited a unique peak wavelength that varied with α composition. Because the peak wavelength in the higher α region was relatively constant, this feature was unique to the fibrils formed under lower α conditions. All these results demonstrated a clear α-dependency, which can be explained by differences in the shape of the energy landscape during fibril formation. Because multiple fluorescence properties were observed only under the lower α-condition with bright and compact aggregates, fibril growth under these conditions proceeds via a multiple equivalent funnel model. Considering that this composition region mimics those in patients with familial AD, our results, which show diversity in β-sheet structure, could be associated with the specific molecular mechanisms in the brains of patients with AD.

## Experimental Procedure

Aβ40 (human, purity 95.0%, Anygen Co., Ltd., Gwangju, Republic of Korea), Aβ42 (human, purity 95.1%, Anygen Co., Ltd., Gwangju, Republic of Korea), ThT (FUJIFILM Wako Pure Chemical Industry, Osaka, Japan), and 1,1,1,3,3,3-hexafluoro-2-propanol (HFIP, purity ≥99.0%, Fujifilm Wako) were purchased and used without further purification. A phosphate-buffered aqueous solution (pH 7.4) containing 100 mM NaCl (Kanto Chemical Co., Inc., purity ≥99.5%, Tokyo, Japan) was used for the sample preparation and measurements.

ThT was dissolved in a phosphate buffer solution by agitation at 40 °C for 5 h in the dark. Aβ (0.5 mg; Aβ40 or Aβ42) was dissolved in 2200 μL of HFIP, left to stand for 1 h, and dispensed (100 μL) into 1.5 mL microcentrifuge tubes. The solvent was evaporated entirely and the Aβ powder was stored at −28 °C until use. Aβ solutions were prepared by dissolving the Aβ powder in 0.02 M NaOH and vortexing for 30 s. Next, phosphate buffer (pH 7.4) containing 100 mM NaCl was added, and the solution was vortexed for 30 s. Nine samples with different α-values were prepared (Table 1) and used for all measurements.

Fibril formation kinetics were monitored using a BioTek Synergy H1Multimode Reader (Agilent Technologies, Santa Clara, CA, USA), with excitation and emission wavelengths of 450 and 485 nm, respectively. Fluorescence intensity from ThT bound to fibrils was monitored over 96 h. From the resulting time–course curves of the ThT fluorescence intensity, the induction period and elongation rate were calculated numerically. First, the time course curves were differentiated. In the first derivative curve, the time corresponding to the initial increase in fluorescence intensity was considered the induction period, while the slope of the region exhibiting a constant increase after this initial increase was defined as the growth rate.

After the kinetics measurements, the sample solution was collected for fluorescence microscopy and fluorescence spectroscopy. Macroscopic morphology was observed using a fluorescence microscope (BX-53, Olympus, Japan), with excitation and emission wavelengths set to 400–440 and 460–550 nm, respectively. Following image acquisition, both the size and brightness of the structures were analyzed using Image Pro analysis software (Media Cybernetics, Inc., USA). Because the aggregates exhibited clear and distinct fluorescence, their boundaries were defined along the interface with the background. The area enclosed within these boundaries was used to measure the size of the aggregates, while the average brightness within the same region was quantified as the fluorescence intensity of the aggregates.

Excitation and emission spectra were acquired using a fluorescence spectrometer (FP-8300, Jasco, Japan). Fluorescence spectra were recorded at an excitation wavelength of 450 nm and an emission wavelength range of 460–650 nm, while excitation spectra were obtained at an emission wavelength of 485 nm with an excitation wavelength range of 400–470 nm. Both the excitation and emission bandwidths were set to 2.5 nm, and the scanning rate was 200 nm/min.

## Supporting Information Available

Data for the experiments performed under a fixed Aβ concentration (PDF)

## Abbreviations

Aβ: amyloid β
AD: Alzheimer’s disease
AFM: atomic force microscopy
NMR: nuclear magnetic resonance
TEM: transmission electron microscopy
ThT: thioflavin T
TICT: twisted internal charge transfer
