# Supplementary material for: Spectral Heterogeneity of Thioflavin T Binding to Aβ42:Aβ40 Mixed Fibrils: Implications for Alzheimer’s Disease Screening
Source: ACS Omega. 2025 Apr 21;10(16):17043–50. doi: 10.1021/acsomega.5c02756 (PMC12044488; doi:10.1021/acsomega.5c02756)
Supplement: Supplementary file 1 — ao5c02756_si_001.pdf [file ao5c02756_si_001.pdf]

Supplementary Information

Spectral Heterogeneity of Thioflavin T Binding to Aβ42:Aβ40 Mixed Fibrils: Implications for Alzheimer's Disease Screening

Kiyo Fukase†, Akane Iida-Adachi†, Hideki Nabika‡,*

†Department of Science, Graduate School of Science and Engineering, Yamagata University, 1-4-12, Kojirakawa, Yamagata 990-8560, Japan

‡Faculty of Science, Yamagata University, 1-4-12 Kojirakawa, Yamagata 990-8560, Japan

Email: nabika@sci.kj.yamagata-u.ac.jp

Data for the experiments performed under a fixed Aβ concentration

The total Aβ protein concentration was fixed at 5 μM, and the experiments were conducted as described in the manuscript.

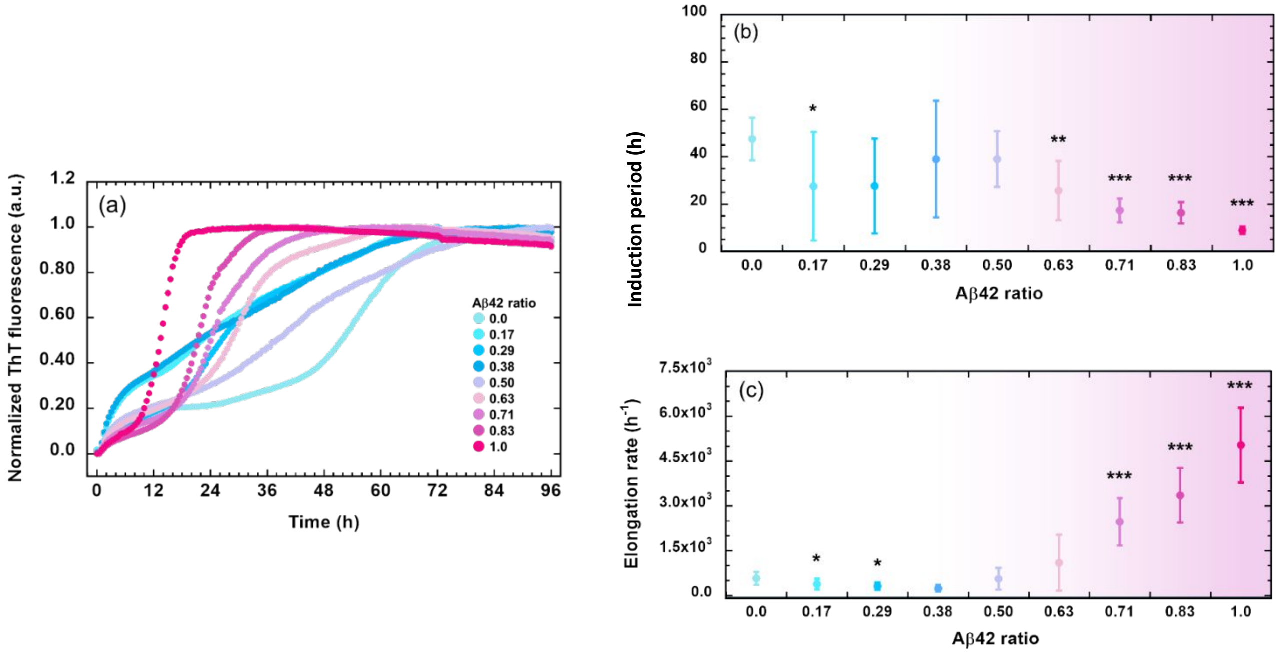

Figure S1. (a) Kinetics of fibril formation at various α values. (b) Induction period and (c) elongation rate as a function of α, derived from the fibril growth curves. Data is represented as mean ± SD. Data was analyzed using the Mann–Whitney U test, with data comparisons for α = 0.0. *p<0.05, **p<0.01, ***p<0.001.

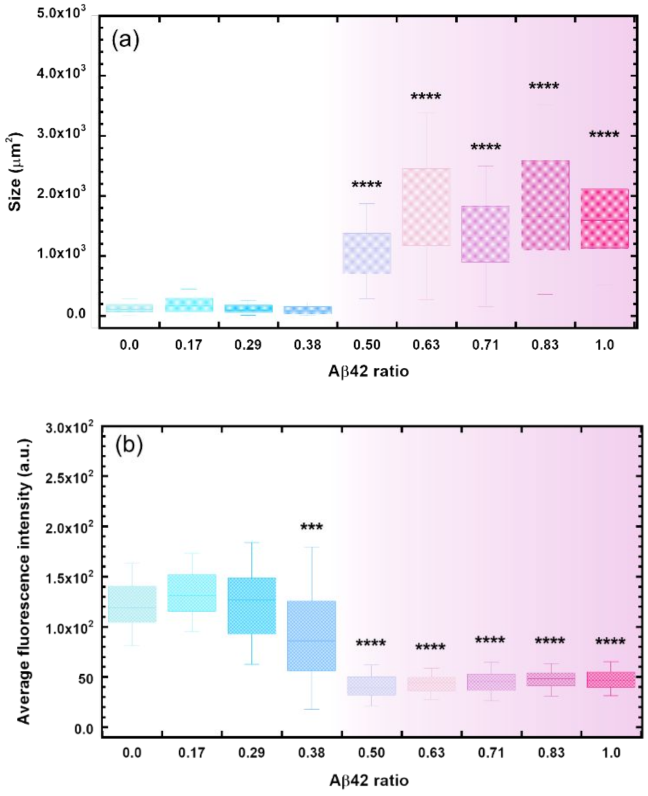

Figure S2. Variation in the (a) size and (b) fluorescence intensity of fibrils as a function of α. Data is represented as mean ± SD. Data was analyzed using the Mann–Whitney U test, with data comparisons for α = 0.0. *p<0.05, **p<0.01, ***p<0.001, ****p<0.0001.

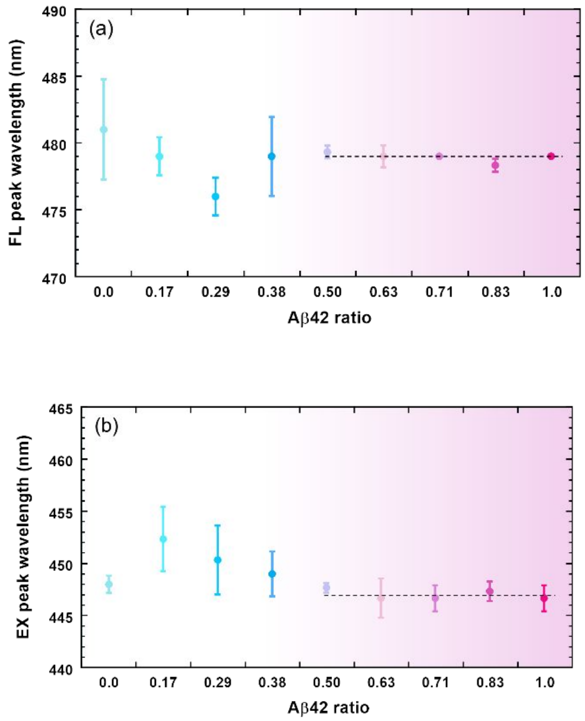

Figure S3. Peak wavelengths of (a) excitation and (b) emission spectra as a function of α. Data is represented as mean ± SD. Data was analyzed using the Mann–Whitney U test, with comparisons between $0 < \alpha < 0.38$ and $05 < \alpha < 1.0$. *$p<0.05$.
